# The Sorghum Gene for Leaf Color Changes upon Wounding (*P*) Encodes a Flavanone 4-Reductase in the 3-Deoxyanthocyanidin Biosynthesis Pathway

**DOI:** 10.1534/g3.115.026104

**Published:** 2016-03-17

**Authors:** Hiroyuki Kawahigashi, Shigemitsu Kasuga, Yuji Sawada, Jun-ichi Yonemaru, Tsuyu Ando, Hiroyuki Kanamori, Jianzhong Wu, Hiroshi Mizuno, Mitsuru Momma, Zui Fujimoto, Masami Yokota Hirai, Takashi Matsumoto

**Affiliations:** * National Institute of Agrobiological Sciences, 2-1-2, Kan-non-dai, Tsukuba 305-8602, Japan; † Faculty of Agriculture, Shinshu University, 8304 Minami-Minowa-mura, Kamiina-gun, Nagano, 399-4598, Japan; ‡ RIKEN Center for Sustainable Resource Science, 1-7-22 Suehiro-cho, Tsurumi-ku, Yokohama, Kanagawa 230-0045, Japan

**Keywords:** apigenin, luteolin, DFR, tan color, gene mapping

## Abstract

Upon wounding or pathogen invasion, leaves of sorghum [*Sorghum bicolor* (L.) Moench] plants with the *P* gene turn purple, whereas leaves with the recessive allele turn brown or tan. This purple phenotype is determined by the production of two 3-deoxyanthocyanidins, apigeninidin and luteolinidin, which are not produced by the tan-phenotype plants. Using map-based cloning in progeny from a cross between purple Nakei-MS3B (*PP*) and tan Greenleaf (*pp*) cultivars, we isolated this gene, which was located in a 27-kb genomic region around the 58.1 Mb position on chromosome 6. Four candidate genes identified in this region were similar to the maize leucoanthocyanidin reductase gene. None of them was expressed before wounding, and only the Sb06g029550 gene was induced in both cultivars after wounding. The Sb06g029550 protein was detected in Nakei-MS3B, but only slightly in Greenleaf, in which it may be unstable because of a Cys252Tyr substitution. A recombinant Sb06g029550 protein had a specific flavanone 4-reductase activity, and converted flavanones (naringenin or eriodictyol) to flavan-4-ols (apiforol or luteoforol) *in vitro*. Our data indicate that the Sb06g029550 gene is involved in the 3-deoxyanthocyanidin synthesis pathway.

Sorghum (*Sorghum bicolor* Moench) is a C4 grass, and is the fifth most commonly grown cereal in the world. It is tolerant to dry environments so is an important crop in the semiarid tropics. Sorghum diverged from maize just 15 million yr ago, and is an important subject of plant genomics research. The sorghum whole genome contains 750 Mb of DNA, or twice as much as the rice genome, and was recently analyzed (Paterson *et al.* 2009).

Sorghum is also a rich source of sorghum-specific natural products. Upon wounding or pathogen invasion, leaves of sorghum plants with the *P* gene (*PP* or *Pp*) turn purple, whereas leaves with the recessive allele (*pp*) turn brown or tan. The *P* gene was initially mapped by Rami *et al.* (1998). Mace and Jordan (2010) combined the data from previous studies and mapped the *P* gene to 58 Mb on chromosome 6, although no homologs of the known genes of the 3-deoxyanthocyanidin synthesis pathway have been detected in this region (Liu *et al.* 2010).

The pigments responsible for this change in color comprise structurally related compounds, 3-deoxyanthocyanidins (luteolinidin and apigeninidin; Dykes and Rooney 2006). They accumulate within inclusions in the epidermal cells as a defense response under pathogen attack (Snyder and Nicholson 1990; Snyder *et al.* 1991). Intriguingly, sorghum leaf color changes upon wounding is closely associated with resistance to foliar diseases (Klein *et al.* 2001; Kasuga *et al.* 2005; Du *et al.* 2010).

In sorghum, anthocyanidins and 3-deoxyanthocyanidins are synthesized by two partially overlapping, competing pathways, although 3-deoxyanthocyanidins are the predominant group in sorghum. Both pathways share the same early steps, which are consecutively catalyzed by phenylalanine ammonia lyase (PAL), chalcone synthase (CHS), and chalcone isomerase (CHI), resulting in the flavanone naringenin. Naringenin enters the anthocyanidin pathway, which employs flavanone-3-hydroxylase (F3H), dihydroflavonol 4-reductase (DFR), and anthocyanidin synthase (ANS). Alternatively, naringenin may enter the 3-deoxyanthocyanidin pathway (Dixon and Paiva 1995; Hipskind *et al.* 1996; Lo and Nicholson 1998).

In the 3-deoxyanthocyanidin synthesis pathway, naringenin is converted into flavan-4-ols (apiforol and luteoforol), and then to 3-deoxyanthocyanidins (apigeninidin and luteolinidin) (Lo and Nicholson 1998; Liu *et al.* 2010). The conversion of naringenin into apiforol is direct, whereas the conversion into luteoforol requires a flavanone intermediate, eriodictyol, which is synthesized from naringenin by flavonoid 3′-hydroxylase (F3′H) (Shih *et al.* 2006).

Naringenin may also enter into flavone (apigenin and luteolin) synthesis pathway by flavanone synthase (FNSII) by converting flavanones to flavone through the formation of 2-hydroxyflavanones (Du *et al.* 2010). The conversion of naringenin into apigenin is direct, whereas the conversion into luteolin requires a flavanone intermediate, eriodictyol. Tan color sorghum cultivars accumulate apigenin, luteolin, or both, instead of the 3-deoxyanthocyanidin (Siame *et al.* 1993; Dykes and Rooney 2006).

Two transcription factors, encoded by the *Y1* and *Tan1* genes, have been detected by a genome-wide association study of sorghum pigmentation (Morris *et al.* 2013). The *Y1* gene controls the sets of structural genes of 3-deoxyantnocyanidin synthesis (Ibraheem *et al.* 2010). The *Tan1* gene has a regulatory function in the anthocyanin and proanthocyanidin synthesis pathways in sorghum seed coat color (Wu *et al.* 2012).

During pathogen-induced 3-deoxyanthocyanidin accumulation in sorghum, the expression of the *PAL* and *CHI* genes is induced, and the respective enzymes are activated, *CHS* and *DFR* genes are upregulated, and the expression of *F3H* and *ANS* genes is strongly suppressed (Hipskind *et al.* 1996; Liu *et al.* 2010). Four DFR genes and one ANS gene have been detected in the sorghum genome (Paterson *et al.* 2009). The potential involvement of DFRs and ANS in the production of flavan-4-ols from flavanones has been tested, but the enzymes that catalyze the last steps of the 3-deoxyanthocyanidin pathway have not been unambiguously identified (Lo and Nicholson 1998; Liu *et al.* 2010).

By looking at genome-wide association signals for the sorghum *P* locus, the most significant SNP (S6_57865283) is detected 260 kb upstream of a large cluster of putative reductase genes. The putative reductase genes are homologous to *Arabidopsis TRANSPARENT TESTA3* and *BANYULS*, and maize *ANTHOCYANINLESS1* (Morris *et al.* 2013).

*TRANSPARENT TESTA 3* and maize *ANTHOCYANINLESS1* encode DFR protein. DFR enzymes of certain plants possess an additional flavanone 4-reductase (FNR) activity. For example, *Lotus japonicus* DFR2, DFR5, and *Malus domestica* DFR have both DFR and flavanone 4-reductase (FNR) activities (Fischer *et al.* 2003; Shimada *et al.* 2005). Flower extracts from *Sinningia cardinalis* have FNR activity in 3-deoxyanthocyanidin synthesis (Stich and Forkmann 1988). *BANYLUS* is a DFR-like protein but encodes an anthocyanidin reductase, which converts anthocyanidins to their corresponding 2,3-*cis*-flavan-3-ols in the proanthocyanidin pathway (Xie *et al.* 2003). Both anthocyanidin reductase and leucoanthocyanidin reductase (LAR) can produce the flavan-3-ol monomers required for formation of proanthocyanin polymers, which is also known as condensed tannin.

In addition, mRNA-seq analysis of target leaf infection in a cultivar BTx623 during the accumulation of 3-deoxyanthocyanidins, a reductase gene, Sb06g029550 was clearly induced among 13 putative LARs in the cluster of putative reductase genes (Mizuno *et al.* 2012).

The objective of this study was to isolate the sorghum gene for leaf color changes upon wounding involved in 3-deoxyanthocyanidin biosynthesis. We used cultivars Nakei-MS3B (purple phenotype, *PP*) and Greenleaf (tan phenotype, *pp*) to identify our gene of interest by genetic mapping with sorghum SSR markers (Yonemaru *et al.* 2009). Using map-based cloning, we found four candidate genes encoding maize LAR homologs. In both cultivars, only one of these genes, Sb06g029550, was induced by leaf cutting. The Sb06g029550 protein was detected only in Nakei-MS3B. Recombinant Sb06g029550 protein had a FNR activity. Sb06g029550 probably converts flavanone to flavan-4-ol in the 3-deoxyanthocyanidin synthesis pathway induced by wounding and pathogen attack. We also found that the loss of function of the Sb06g0129550 gene is associated with the tan phenotype in sorghum.

## Materials and Methods

### Plant materials and phenotyping

Sorghum [*Sorghum bicolor* (L.) Moench] cultivars Nakei-MS3B (*PP*) and Greenleaf (*pp*) were crossed to establish a mapping population. F_3_ and F_5_ generations were grown and tested at Shinshu University (Minowamura, Nagano, Japan) in 2009 and 2010. High-density genetic mapping of F_3_ progeny was performed. To determine the phenotypes (purple or tan), F_3_ progeny were grown in pots at 28.5–30° in a greenhouse, and leaves of 2-month-old plants were punched. The color of the damaged leaves was examined 7 days later. In the field, naturally damaged leaves were examined in August. Four accessions with the tan phenotype (JP501, JP588, JP43764, and JP43800) were obtained from the National Institute of Agrobiological Sciences (NIAS) sorghum core collection (Shehzad *et al.* 2009). The passport and evaluation data for the four accessions can be reviewed at the NIAS Genebank website (http://www.gene.affrc.go.jp/about_en.php).

### Marker development and genetic mapping

The progeny used for genetic mapping included 175 F_5_ recombinant inbred lines, and 1142 F_3_ generation plants. Sorghum simple sequence repeat (SSR) markers designed previously (Yonemaru *et al.* 2009), and the other polymorphism markers listed in Supplemental Material, Table S1 were used. Genomic DNA was isolated using the cetyltrimethylammonium bromide (CTAB) method (Murray and Thompson 1980), and specific fragments were amplified by PCR. SSR genotyping was performed in 10-µl reaction mixtures containing genomic DNA (2 ng/µl), forward and reverse primers (6 nmol each), dNTPs (75 μM each), and 1 U of *Taq* DNA polymerase (Promega). The PCR program used 35 cycles of 94° for 20 sec, 55° for 30 sec, and 72° for 3 min. The PCR products were analyzed in 3% agarose gels. The markers selected after bulked segregant analysis were mapped in the entire population with MAPMAKER v. 3.0 software (Lander *et al.* 1987). The F_2_ intercross algorithm and default linkage criteria (LOD score 3.0 and a maximum distance of 50 cM) were applied. The Kosambi mapping function was used to calculate genetic distances.

BAC libraries constructed from DNA of young leaves contained 39,267 clones (Nakei-MS3B, average insert size of 134 kb), or 55,000 clones (Greenleaf, 124 kb). The libraries were prepared using conventional methods, including partial DNA digestion with *Hin*dIII, size fractionation of high-molecular-weight DNA by pulsed-field gel electrophoresis (CHEF, Bio-Rad Laboratories), vector ligation (pIndigo BAC-5, Epicentre Biotechnologies), and transformation into *Escherichia coli* (strain DH10B). Positive BAC clones covering the region of the *P* gene were screened from each library using tightly linked DNA markers and PCR. Identified BACs were shotgun-sequenced (∼10× coverage) and assembled as described previously (Sasaki *et al.* 2002; Wu *et al.* 2003). The BAC clones MS3B-70D15 (155 kb) from Nakei-MS3B and GL-25I05 (87.4 kb) from Greenleaf were identified with the CA29490 and SB25792 PCR markers (Table S1). The sequences of candidate genes were obtained from the sorghum genome database (http://www.plantgdb.org/SbGDB). Homology of the candidate genes was searched by TBLASTX (https://blast.ncbi.nlm.nih.gov/Blast.cgi).

### Phylogenetic analysis

Protein sequences were aligned with CLUSTALW (Thompson *et al.* 1994) at the DNA Data Bank of Japan website (DDBJ; http://www.ddbj.nig.ac.jp/search/clustalw-e.html). A phylogenetic tree was generated with NJplot software (http://doua.prabi.fr/software/njplot; Perriere and Gouy 1996).

### RT-PCR

Mature leaves of 2-month-old plants were cut into 3-mm strips and placed on 2% agar plates at 28°. The leaf strips were analyzed at different time points as indicated in the figure legends. Total RNA was extracted with a Get Pure RNA Kit (Dojindo). First-strand cDNA was synthesized from RNA (1 µg) in a 20-µl reaction mixture with a TaKaRa RNA PCR kit (AMV) v. 3.0 (TaKaRa Bio, Inc.). PCR was performed as described previously (Kawahigashi *et al.* 2011), with initial denaturation at 94° for 5 min; 30–35 cycles of 94° for 20 sec, 55° for 30 sec, and 72° 1.5 min; and a final extension at 72° for 5 min. PCR products (4 µl) were analyzed in 2% agarose gels. PCR primers are listed in Table S2. SbActin (Sb03g040850) was used as positive control.

Quantitative RT PCR (qRT-PCR) was carried out using Mx3000P (Stratagene Products Division, Agilent Technologies) with KOD SYBR qPCR Mix (Toyobo) according to the manufacturer’s recommendations. The gene transcripts were amplified with each specific primer pair (Table S1 and Table S2 for Sb06g029550). The value for each genes was normalized using SbActin as an internal standard.

### Recombinant Sb06g029550 production and modeling

To produce protein for antibody production, the DNA fragment encoding Sb06g029550 was amplified using Phusion DNA polymerase (New England Biolabs) with specific primers (Table S1). The fragment was subcloned into the pET45b expression vector (Merck-Novagen), and the plasmid was transformed into *E. coli* strain BL21 (DE3). Expression was induced with 0.5 mM isopropyl β-d-thiogalactoside; the culture was incubated at 25° overnight. The protein was purified by nickel-chelating chromatography on a 5-ml His-Trap Fast Flow column (GE Healthcare, Buckinghamshire, UK). The molecular weight of the recombinant protein was estimated by SDS-PAGE to be 40 kDa, which agreed well with the predicted value (38 kDa). The protein was used for generation of rabbit polyclonal antibody against Sb06g029550 by Operon Biotechnology, Ltd. The Surface model of sorghum Sb06g029550 protein was created with the SWISS-MODEL web server (Arnold *et al.* 2006) by homology modeling on the basis of the crystal structure of grape dihydroflavonol 4-reductase (Protein Data Bank code 3C1T; Trabelsi *et al.* 2008).

### Western blotting

Soluble protein fractions from leaf strips incubated on agar plates were prepared as previously reported (Kawahigashi *et al.* 2005). Protein from soluble fraction (30 µg per lane) was analyzed, and recombinant Sb06g029550 protein (described below) was used as a positive control. Rabbit polyclonal antibody against Sb06g029550 was generated by Operon Biotechnology, Ltd., using the recombinant protein as an antigen. Sb06g029550 protein was detected by the rabbit antibody, and an ECL protein detection system (GE Healthcare Japan) according to the manufacturer’s instructions.

### Thin-layer chromatography (TLC) analysis

Damaged leaves were harvested from the field. Tissues were incubated in methanol containing 0.1% HCl overnight at 4°, then in 1 *N* HCl at 100° for 1 hr (Shimizu *et al.* 1996); 3-deoxyanthocyanidins were extracted with small amount of isoamyl alcohol by shaking at room temperature. The isoamyl alcohol was evaporated and the residue was dissolved in methanol containing 0.1% HCl and separated on TLC Cellulose F plates (Merck, Darmstadt, Germany) in HCl: acetic acid: water (3:30:60, v/v/v).

### Analysis of 3-deoxyanthocyanidin accumulation

Leaf strips of 2-month-old plants were placed on 2% agar plates at 28°, and sampled 0, 2, 4, and 6 d after cutting. For the detection of luteolin from Greenleaf cultivar, leaf strips were sampled 3 d after cutting. Leaf strips (0.1 g fresh weight) were incubated in methanol containing 0.1% HCl overnight at 4°, and the absorbance at 493 nm was measured on a DU640 spectrometer (Beckman).

The extracts prepared as TLC analysis, were subjected to high-performance liquid chromatography (HPLC)-mass spectrometry (MS) (Hewlett-Packard 1100MSD). The solvent system for HPLC was 0.1% acetic acid in water, and a linear methanol gradient was applied (0–100%, 25 min). Metabolites were separated at a flow rate of 1.0 ml/min on a 4.6-mm × 150-mm Comosil 5C18-AR column (Nacalai Tesque), and detected at a wavelength of 320 nm for flavones, and 530 nm for 3-deoxyanthocyanidins. The conditions for MS were as follows: atmospheric pressure ionization–electrospray mode; capillary voltage, 3500 V; focus voltage, 30 V; nebulizer pressure, 25 PSI; dry gas flow, 10 liter/min; dry gas temperature, 350°; polarity, positive; fragmentor voltage, 60 V.

Naringenin and apigenin were purchased from Wako Chemical Co., Ltd., luteolin from Cayman Chemical Company, apigeninidin from Fluka Sigma-Aldrich, and luteolinidin from ChromaDex, Inc.

### In vitro assay of recombinant Sb06g029550 protein activity

To obtain N-terminally His-tagged Sb06g029550 for enzymatic assays, cDNA was subcloned into the *Nhe*I–*Xho*I sites of a Gateway Nova pET-53-DEST vector (Merck KGaA). The plasmid was transformed into *E. coli* strain BL21 Star (DE3) pLysS (Life Technologies). Expression was induced by incubating the culture at 18° for 48 hr and then at 30° for 5 hr. Then, 2 mg of protein was purified from the cell pellet (5 g) with an IMAC system (Takara). The reaction mixtures (500 μl) for assay contained 0.1 M potassium phosphate buffer (pH 7.0), 1 mg of NADPH, and 200 μg of a substrate [naringenin, eriodictyol, dihydroquercetin (ChromaDex, Inc.), or (±)-taxifolin (dihydrokaempferol) (ChromaDex, Inc.)]. The recombinant protein (250 μg) was added, and the mixture was incubated for 12 hr at 30°. The reaction was terminated by extraction with ethyl acetate (500 μl). Following evaporation of 200-μl extracts, residues were dissolved in 2 *N* HCl in 50% methanol, incubated for 1 hr at 80°, dried, and dissolved in 200 μl of 0.1% formic acid in water. The reaction mixtures were acid-treated to convert the direct reaction products, flavan-3, 4-diol, and flavan-4-ol, to their respective anthocyanidin or 3-deoxyanthocyanidins, and analyzed by LC-MS/MS (Sawada *et al.* 2009).

### Data availability

The authors state that all data necessary for confirming the conclusions presented in the article are represented fully within the article. Sorghum strains are available upon request. Sequence data are available at GenBank and the accession numbers are AB671764 for Nakei-MS3B and AB671765 for Greenleaf Figure S1 contains SDS-PAGE data. Figure S2 contains the repeat sequence and flanking sequences of a large insert in the Sb06g029550 allele in accessions JP501 and JP43800. Figure S3 contains expression of genes associated with secondary metabolism of 3-deoxyanthocyanidins, anthocyanindins or flavones in Nakei-MS3B. Expression data are available at http://www.ncbi.nlm.nih.gov/pmc/articles/PMC4219097/. Figure S4 contains surface model of sorghum Sb06g029550 protein. Table S1 and Table S2 contain PCR primer sequences used for this study.

## Results

### Purple and tan phenotypes in sorghum

**Figure 1:**
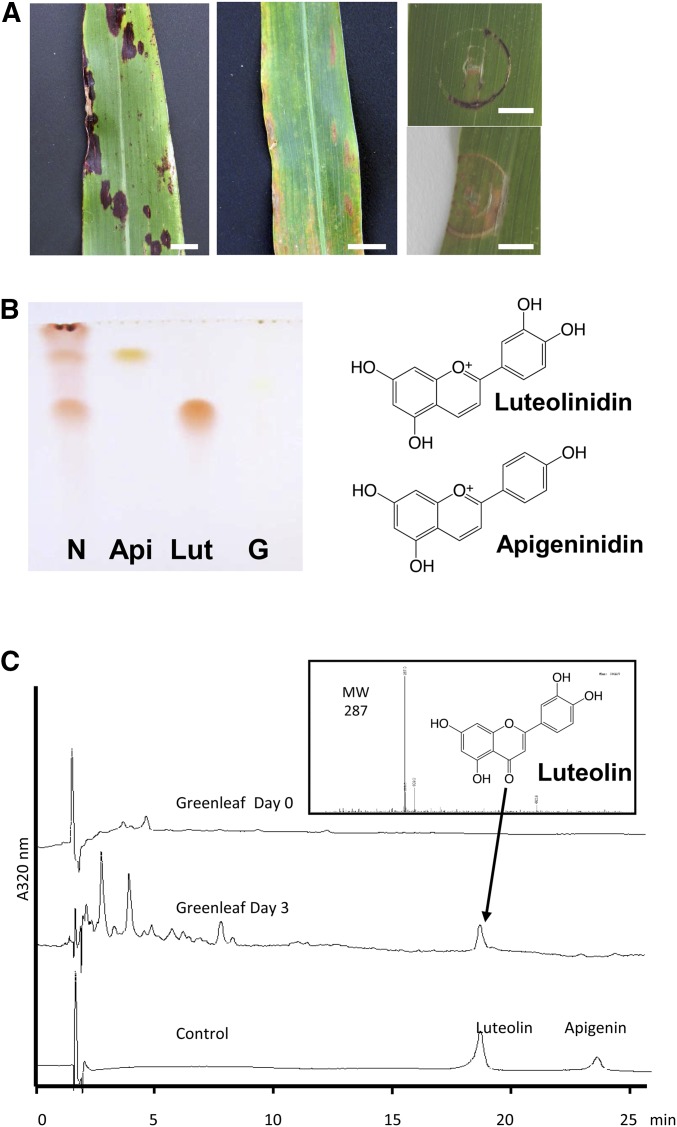
Phenotypes of sorghum cultivars Nakei-MS3B (purple) and Greenleaf. Natural leaf damage (left and middle panels) and leaf punching (upper right panel; NakeMS3B, lower right panel; Greenleaf). Scale bars, 0.5 cm. (B) TLC analysis of 3-deoxyanthocyanidins extracted from damaged leaves. N, Nakei-MS3B; Api, apigeninidin standard; Lut, luteolinidin standard; G, Greenleaf. (C) HPLC-MS analysis of leaf extracts of Greenleaf (d 3 after leaf cutting).

After natural leaf damage or leaf punching, Nakei-MS3B exhibited typical purple leaf lesions (Figure 1A), whereas those of Greenleaf were tan color (Figure 1A). In TLC analysis, apigeninidin and luteolinidin were detected in the damaged leaves of Nakei-MS3B, but not in those of Greenleaf (Figure 1B). In HPLC-MS analysis, a major peak at 18.3 min was detected on d 3 in Greenleaf. Comparison of its retention time and mass spectrum (MW 287) with those of the standard identified this peak as luteolin (Figure 1C).

### Mapping of the gene for leaf color changes upon wounding and analysis of four candidate genes

**Figure 2:**
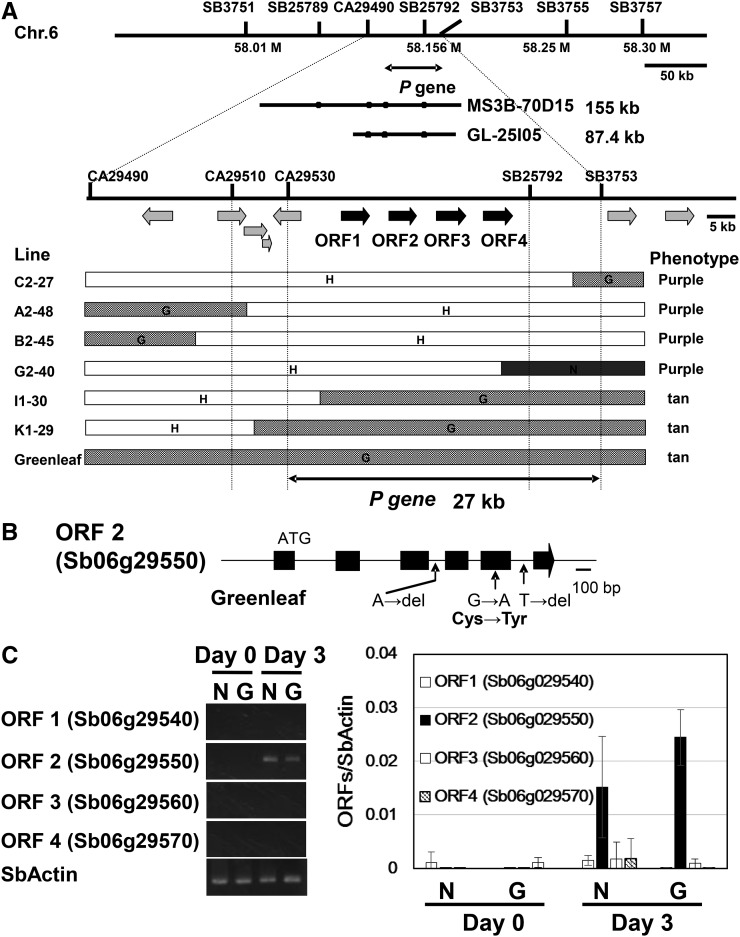
Positional cloning of the gene for leaf color changes upon wounding (*P*). (A) The *P* gene was mapped to a region on chromosome 6 comprising approximately 27 kb between the markers CA29530 and SB25792. The BAC clones MS3B-70D5 (from Nakei-MS3B) and GL-25I05 (from Greenleaf) were isolated from BAC libraries by PCR screening with the CA29490 and SB25792 markers. Four candidate ORFs found in this region in the sorghum database (http://www.plantgdb.org/SbGDB) are shown as black arrows, with flanking ORFs shown as gray arrows. High-resolution mapping detected six recombination events. G, regions from Greenleaf; N, regions from Nakei-MS3B; H, heterozygous regions. (B) Schematic diagram of the Sb06g029550 gene. Exons are shown as black boxes. Positions of mutations in Greenleaf in comparison with Nakei-MS3B are shown below the gene structure. Greenleaf Sb06g029550 has a point mutation that causes an amino acid change (Cys252Tyr). (C, left) RT-PCR analysis of the expression of candidate genes in Nakei-MS3B (N) and Greenleaf (G) on day 0 or day 3 after leaf cutting. SbActin, positive control. (C, right) qRT-PCR analysis of the expression of candidate genes in Nakei-MS3B (N) and Greenleaf (G) on day 0 or day 3 after leaf cutting. The vertical axis shows relative transcripts levels normalized to SbActin. The mean quantified values ± SD for three replicates are shown.

We performed whole-genome mapping by screening 175 F_5_ recombinant inbred lines from a cross between Nakei-MS3B and Greenleaf, and mapped the *P* locus between SSR markers SB3751 (58.01 M) and SB3764 (58.68 M) on chromosome 6. For further mapping, we screened 64 of the 1142 F_3_ seedlings for recombination events between these markers, and localized the gene of interest to a 27-kb region (Figure 2A). A search of the sorghum genome database using the 27-kb region as a query revealed four open reading frames (ORFs) in this region: ORF 1, Sb06g029540; ORF 2, Sb06g029550; ORF 3, Sb06g029560; and ORF 4, Sb06g029570. All of them showed similarity to *Zea mays* LAR (ACG28358) in TBLASTX searches (96%, 95%, 90%, and 84% similarity, respectively). We compared the Nakei-MS3B and Greenleaf sequences of these four genes, including their 2-kb promoter regions, and 0.5 kb of their 3′ UTRs. Genes Sb06g029540 and Sb06g029560 were identical in both cultivars except for a deletion in the 3′ UTR of Sb06g029560; therefore, we excluded them as candidates. The amino acid sequences encoded by Sb06g029550 and Sb06g029570 of Nakei-MS3B were identical to those available for sorghum cultivar BTx623 (*PP*). The Sb06g029550 gene contained six exons in a total genomic sequence of 2259 bp, including 10,325 bp of coding sequence (344 amino acids; MW 37.8 kDa). In Greenleaf Sb06g029550, cysteine 252 was substituted for tyrosine (Figure 2B). There were two deletions (A and T) within homopolymer repeats (10A and 23T) in two introns in Greenleaf (Figure 2B). Sb06g029570 in Greenleaf also had a 1-nt deletion, causing a frameshift in the coding region.

RT-PCR analysis showed that none of the genes was expressed in cut leaves on d 0, and only Sb06g029550 was induced in the leaves of both parental cultivars on d 3 after cutting (Figure 2C, right). The strong induction of ORF2 (Sb06g029550) was also confirmed by qRT-PCR (Figure 2C, left).

These data suggested that Sb06g029550 (accession numbers are AB671764 for Nakei-MS3B and AB671765 for Greenleaf) was the most promising candidate for the gene responsible for the color phenotype. Therefore, we chose this gene for further analysis.

### Time course of 3-deoxyanthocyanidin synthesis and Sb06g029550 expression in Nakei-MS3B and Greenleaf

**Figure 3:**
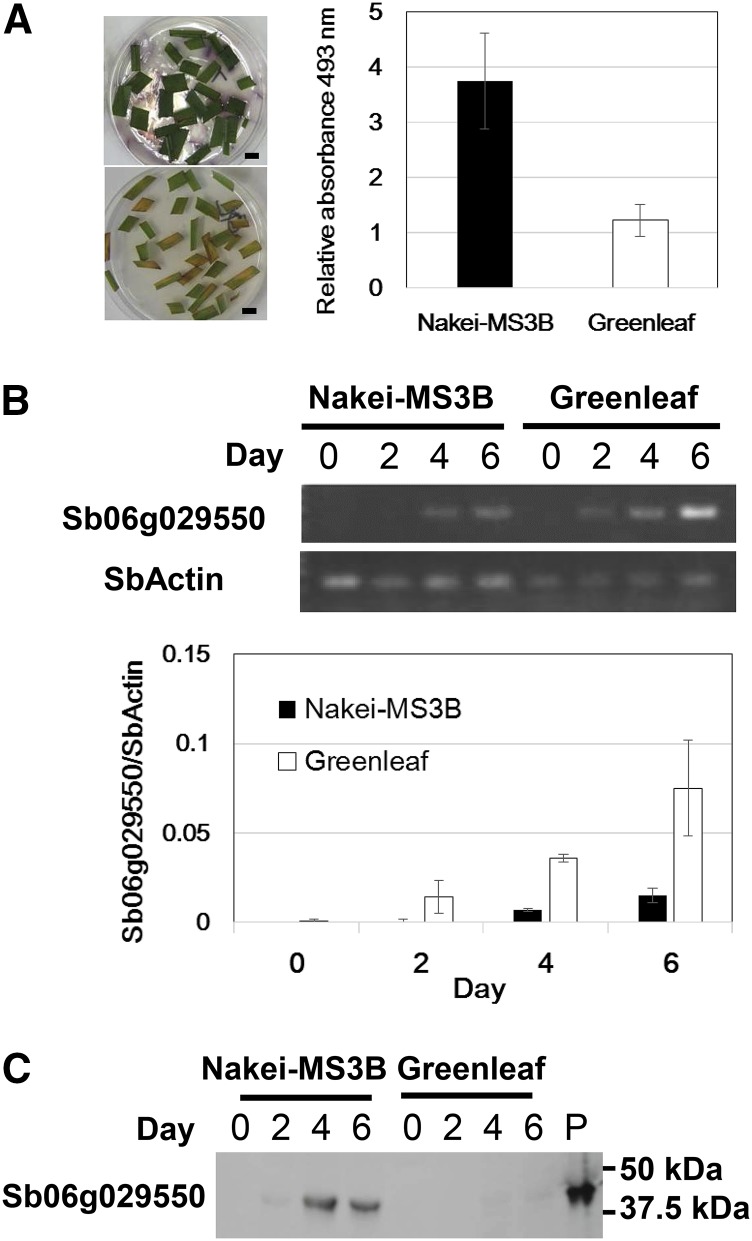
Time course of Sb06g029550 expression and 3-deoxyanthocyanidin induction in leaf strips of Nakei-MS3B and Greenleaf during a 6-d incubation on agar plates. (A) Leaf strips from Nakei-MS3B (upper left panel) and Greenleaf (lower left panel) on d 6. Scale bar, 1 cm. Relative absorbance of 437 nm by spectrophotometric measurement of 3-deoxyanthocyanidins extracted in methanol containing 1% HCl on day 6 (right panel). (B) Time course of detection of the Sb06g029550 transcript by RT-PCR and qRT-PCR. SbActin, positive control. The condition of qRT-PCR is described in Figure 2D. (C) Time course of detection of Sb06g029550 protein in the soluble fraction by Western blotting. P, His-tagged recombinant protein (positive control).

We analyzed the time course of 3-deoxyanthocyanidin synthesis and Sb06g029550 expression in leaf strips kept on agar plates. By d 6, Nakei-MS3B leaf strips were purple, whereas those of Greenleaf were brown (tan color). The pigments, 3-deoxyanthocyandins were induced in Nakei-MS3B but not in Greenleaf (Figure 3A). Induction of Sb06g029550 was detected 2 d after cutting, at least for Greenleaf (Figure 3B). Both cultivars showed induction of Sb06g029550 mRNA 4 d after cutting. It also shows that expression was higher in Greenleaf than in Nakei-MS3B. In Western blot analysis, a polyclonal antibody against Sb06g029550 detected a band of the expected molecular weight in Nakei-MS3B on d 4 and d 6. The protein band was present in Greenleaf at a much lower level than in Nakei-MS3B (Figure 3C and Figure S1A); the amino acid substitution (Cys252Tyr) in Greenleaf Sb06g029550 could increase protein instability and lead to degradation. These data are compatible with the role of Sb06g029550 in determining the color phenotype.

### Analysis of Sb06g029550 in four sorghum accessions with the tan phenotype

**Figure 4:**
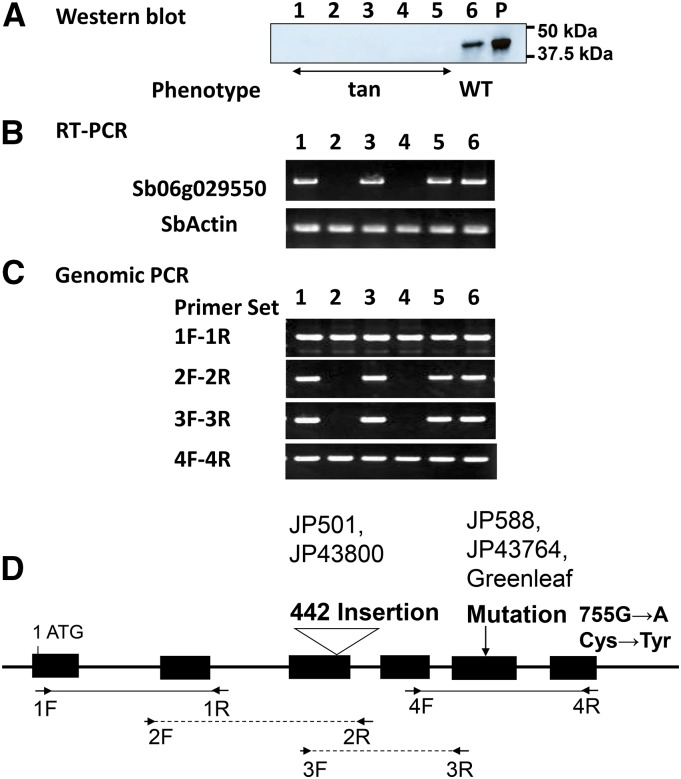
Analysis of Sb06g029550 in sorghum accessions with the tan phenotype. (A) Detection of the Sb06g029550 protein by Western blotting in soluble protein of leaf strips 6 d after cutting. (A–C) Four accessions with the tan phenotype obtained from the NIAS sorghum core collection are marked as 1, JP43764; 2, JP43800 3, JP588; 4, JP501; in addition to 5, Greenleaf; 6, Nakei-MS3B; P, recombinant protein (positive control). (B) Detection of the Sb06g029550 transcript by RT-PCR. SbActin, positive control. (C) Detection of the Sb06g029550 gene by genomic PCR. (D) Exon–intron structure of the Sb06g029550 gene. Positions of genomic PCR primers, a large insert, and a point mutation are shown.

To ascertain the association between Sb06g029550 and the color phenotype, we also analyzed four sorghum accessions with the tan phenotype (Figure 4 and Figure S1B). The Sb06g029550 protein was detected only in Nakei-MS3B, not in any of these accessions or Greenleaf (Figure 4A, lanes 1–5). The Sb06g029550 transcript was detected in JP43764 and JP588 (Figure 4B, lanes 1 and 3), but not in JP43800 and JP501 (Figure 4B, lanes 2 and 4). Genomic PCR detected a single band in JP43764 and JP588 (Figure 4C, lanes 1 and 3), but no band in JP43800 and JP501 (Figure 4C, lanes 2 and 4). Sequencing of the flanking region revealed an insertion (> 4 kb) at 452 bp from the mRNA start codon in JP43800 and JP501 (Figure S2). The other two accessions, JP43764 and JP588, had the same mutation as Greenleaf (G > A at 755 bp), leading to the amino acid substitution Cys252Tyr (Figure 4D).

### In vitro assay of recombinant Sb06g029550 protein activity

**Figure 5:**
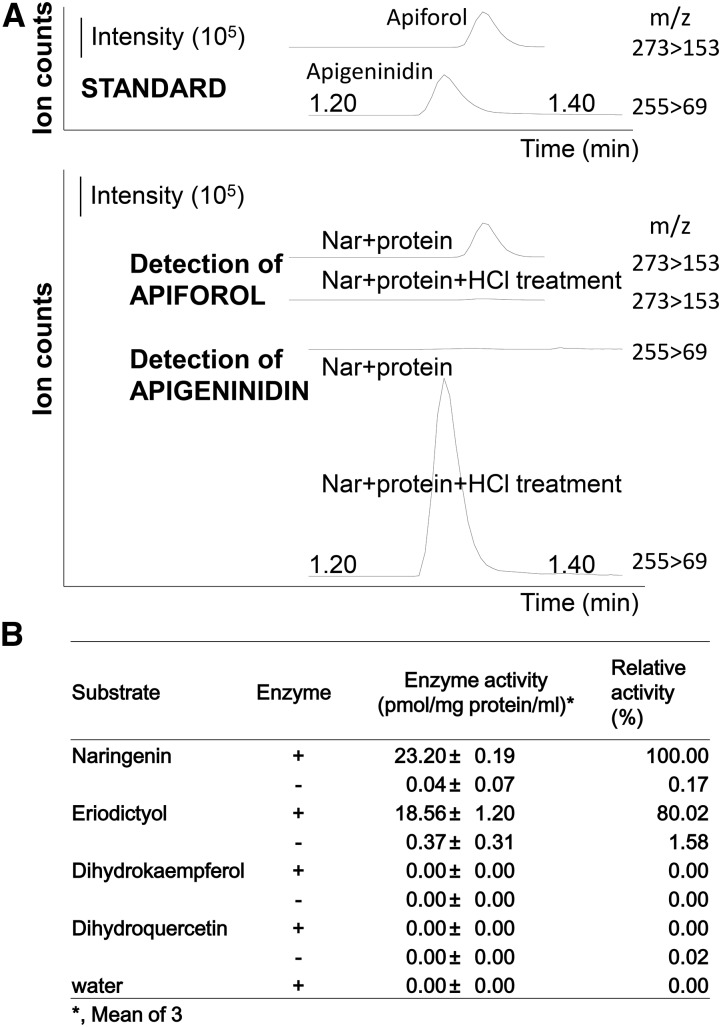
*In vitro* assay of Sb06g029550 activity by HPLC-MS/MS. (A) The substrate naringenin (flavanone) was incubated with recombinant Sb06g029550 protein. Apiforol (flavan-4-ol) was detected as a product (Nar+protein). Apiforol was converted to apigeninidin with HCl (Nar+protein+HCl treatment). Apiforol and apigeninidin standards were detected as precursor ions, 273 *m/z* and 255 *m/z*, and product ions, 153 *m/z* and 69 *m/z*, respectively. (B) Summary of Sb06g029550 activity.

Recombinant Sb06g029550 purified from *E. coli* was incubated with flavanones (naringenin and eriodictyol), and products were analyzed by HPLC-MS/MS (Figure 5). The protein had a strong flavanone 4-reductase activity, and produced a flavan-4-ol (apiforol) from naringenin. HCl treatment was required to convert apiforol to apigeninidin (Figure 5, A and B). Sb06g029550 was also able to use eriodictyol (Figure 5B), a precursor of the flavan-4-ol luteoforol. No products were detected in the absence of Sb06g029550, or in the presence of heat-inactivated enzyme. Sb06g029550 did not use dihydroflavonol, dihydrokaempferol, or dihydroquercetin as substrates. These results showed that Sb06g029550 had an FNR activity (EC 1.1.1.234), but no DFR activity (Figure 5B).

### Genes activated in response to wounding

**Figure 6:**
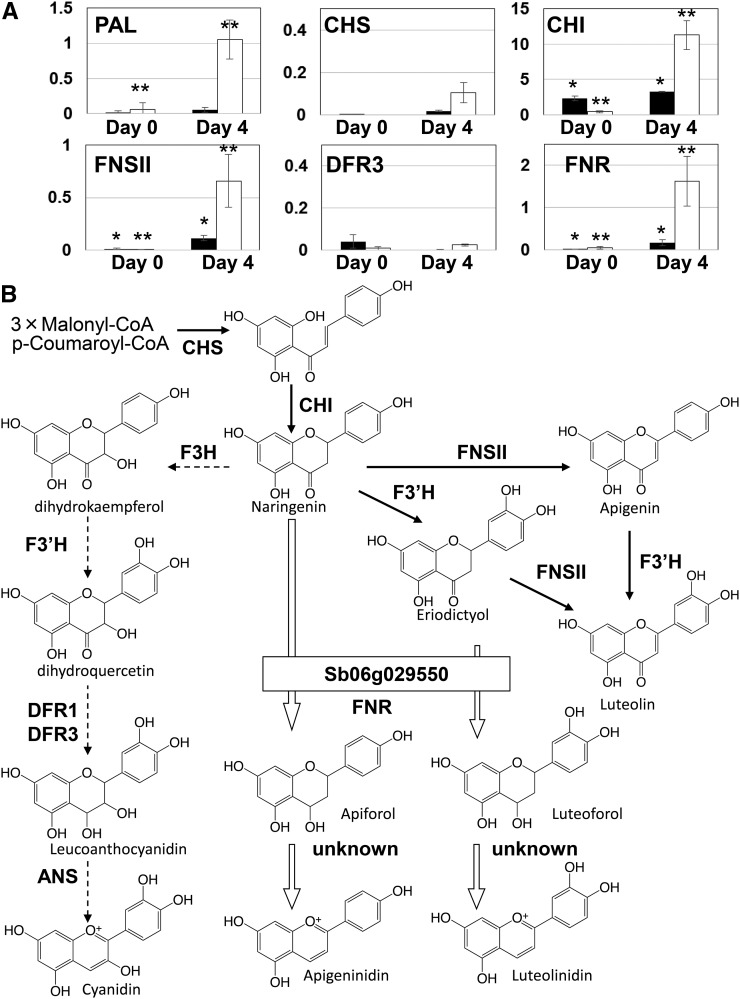
(A) Expression of 3-deoxyanthocyanidin synthesis-related genes after leaf cutting by qRT-PCR analysis. Black bars are Nakei-MS3B and white bars are Greenleaf. The vertical axis shows relative transcripts levels normalized to SbActin. The mean quantified values ± SD for three replicates are shown. Both * and ** show the significant difference after leaf cutting by *t*-test (*P* < 0.05). (B) A schematic diagram of the anthocyanidin, 3-deoxyanthocyanidin, and flavone biosynthesis pathways in damaged sorghum leaves. Bold black arrows indicate steps common to Nakei-MS3B and Greenleaf. White arrows indicate steps that occur in Nakei-MS3B but not in Greenleaf. Dotted arrows indicate the anthocyanidin pathway, which is mediated by enzymes the transcripts of which are not upregulated by wounding in both cultivars. PAL, phenylalanine ammonia lyase; CHS, chalcone synthase; CHI, chalcone isomerase; FNSII, flavone synthase II; FNR, flavanone 4-reductase; DFR, dihydroflavonol 4-reductase; F3H, flavanone 3-hydroxylase; ANS, anthocyanidin synthase; F3′H, flavonoid 3′-hydroxylase.

We analyzed the expression patterns of genes related to the synthesis of 3-deoxyanthocyandin, anthocyanidin, and flavones in Nakei-MS3B and Greenleaf upon leaf cutting (Figure 6A). At 4 d after cutting, *CHI*, *FNR* (Sb06g029550), and *FNSII* were significantly (*P* < 0.05, *t*-test) induced in both cultivars. On both days, *DFR3* was expressed at low level in both cultivars. *DFR1*, *F3H1*, *F3H2*, and *ANS* were not expressed in either cultivar (data not shown). A schematic diagram of the anthocyanidin, 3-deoxyanthocyanidin, and flavone biosynthesis pathways is shown in Figure 6B. We also confirmed that the induction of the *PAL*, *CHS*, *FNR*, *DFR3*, and *FNSII* genes and no expression of F3H1 or ANS in Nakei-MS3B upon leaf cutting by mRNA-seq analysis (Figure S3, Mizuno *et al.* 2014).

### Phylogenetic analysis

**Figure 7:**
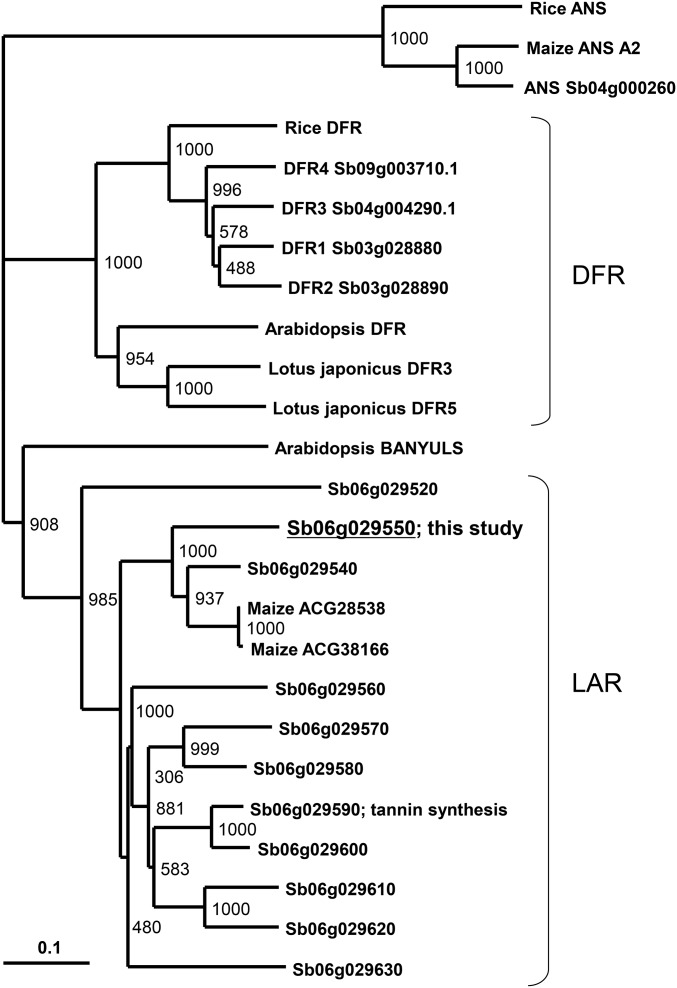
Phylogenetic tree of LAR, ANS, and DFR. Protein sequences were aligned using CLUSTALW, and the phylogenetic tree was generated with NJplot. Bootstrap values (out of 1000 replicates), shown at the nodes, were used to assess the robustness of the tree. Accession numbers are as follows. Rice (*Oryza sativa*): ANS, CAA69252; DFR, AB003496. Maize (*Zea mays*) ANS_A2, NP_001106074. *Arabidopsis thaliana*: DFR, NM_123645, BANYULS, AF092912. *Lotus japonicus*: DFR3, AB162111; DFR5, AB162114.

We performed phylogenetic analysis of 11 LARs (Sb06g029540–Sb06g029630) encoded in a gene cluster on chromosome 6, ANSs, and DFRs (Figure 7). All 11 sorghum LARs clustered in a clade distinct from the DFR clade. Sb06g029550 had FNR activity, clustered with maize LAR, and both were in the same clade as *Arabidopsis* BANYULS. Interestingly, both *Lotus japonicus* DFRs have FNR activity in addition to DFR activity (Shimada *et al.* 2005), were clustered with DFRs, which is a different clade from the LARs. ANS proteins, the members of the 2-oxoglutarate-dependent oxygenase family (Nakajima *et al.* 2001; Winkel-Shirley 2001), were clearly clustered in a different clade. These results indicate that Sb06g029550 is more closely related to DFRs than to ANSs.

## Discussion

In this study, using high-resolution genetic and physical mapping, analysis of the mRNA and protein levels and enzymatic assays, we identified sorghum Sb06g029550 as the *P* gene responsible for leaf color changes upon wounding. Cultivars with the tan phenotype did not produce the Sb06g029550 protein; in some of them (JP501 and JP43800), the gene was disrupted by an insertion, indicating that this enzyme is needed for the purple phenotype. Greenleaf has the Sb06g029550 transcript, but a single nucleotide substitution in the coding region (also present in JP43764 and JP588) leads to an amino acid substitution (Cys252Tyr). According to a three-dimensional homology model of the Sb06g029550 protein, this Cys residue does not seem to be involved in a disulfide bond or the catalytic site, but is located in a hydrophobic cluster inside the protein (Figure S4). Tyr residues are more bulky than Cys residues, and this amino acid substitution may cause protein instability and degradation. The mRNA expression of Sb06g029550 was higher in Greenleaf than in Nakei-MS3B, which indirectly supports the instability hypothesis of the Sb06g029550 protein in Greenleaf. Two deletions (A and T) within homopolymer repeats (10A and 23T) in two introns may affect intron–exon recognition, and result in defects in precursor mRNA formation or splicing, but this is unlikely because full-length mRNA was detected.

Recombinant Sb06g029550 had specific FNR activity to produce flavan-4-ols from flavanones, but had no DFR activity. In contrast, both SbDFR1 and SbDFR3 have low enzymatic activity *in vitro* to produce flavan-4-ols from flavanones (naringenin and eriodictyol), but high DFR activity to produce dihydroflavonol from dihydrokaempferol and dihydroquercetin (Liu *et al.* 2010). Only cyanidin, a product of the anthocyanidin pathway, was detected by complementation analysis in an *Arabidopsis* DFR-deficient line, tt3 with SbDFR3; this led to the conclusion that SbDFR3 prefers dihydroflavonols as substrates, and may be involved mainly in anthocyanidin synthesis in sorghum (Liu *et al.* 2010). Thus, Sb06g029550 may be involved mainly in 3-deoxyanthocyanidin synthesis in sorghum.

A multi-enzyme complex may be involved in flavonoid biosynthesis in *Arabidopsis* (Winkel 2004). Thus, the sorghum pathogen/stress-inducible flavonoid enzymes may form a protein complex that facilitates the conversion of naringenin to flavan-4-ols and 3-deoxyanthocyanidins. Although it would be desirable to generate transgenic Sb0g029550 sorghum plants for complementation assays, low transformation efficiency has so far hampered our efforts; development of a higher-efficiency system for sorghum transformation is needed.

Naringenin is a competitive substrate for F3H, F3′H, Sb06g029550 (FNR), and FNSII (Figure 6). The absence of F3H transcripts in both cultivars upon leaf cutting suggests that these genes are induced by different stimuli. In Nakei-MS3B, naringenin could be a substrate for Sb06g029550 and F3′Hs (Shih *et al.* 2006). The expression patterns of the genes involved in 3-deoxyanthocyanin synthesis in Nakei-MS3B upon wounding were similar to those in BTx623 upon leaf infection; the latter cultivar also accumulated 3-deoxyanthocyanidins (Mizuno *et al.* 2012). Sb06g029550 was clearly induced among 13 putative LARs (Mizuno *et al.* 2012). In Greenleaf, the flavone luteolin accumulated in the leaves, suggesting that naringenin entered a pathway that includes F3′H and FNSII (Figure 6). This result is consistent with previous reports that the formation of flavan-4-ols is blocked in the synthesis of pigments in tan plants (Siame *et al.* 1993, Dykes *et al.* 2005). In both Greenleaf and Nakei-MS3B, the ANS gene was not induced; therefore, ANS is not responsible for the conversion of flavan-4-ols to 3-deoxyanthocyanidins (the final step in 3-deoxyanthocyanidin synthesis). The enzyme responsible for this step remains to be identified.

The Sb06g029550 showed sequence similarity to LARs. Sb06g029550 may have functionally diverged from the other LARs, and they may function at different steps of the flavonoid biosynthesis pathway. The Sb06g029590 gene is induced during tannin synthesis in the seed coat development, and it is involved in the proanthocyanidin synthesis pathway (Wu *et al.* 2012).

In summary, our findings indicate that the sorghum gene Sb06g029550 is responsible for changes in sorghum leaf color upon wounding. The Sb06g029550 protein has FNR activity *in vitro*, and may function in the 3-deoxyanthocyanidin synthesis pathway.

## Supplementary Material

Supplemental Material
