# Supplementary material for: The Sorghum Gene for Leaf Color Changes upon Wounding (P) Encodes a Flavanone 4-Reductase in the 3-Deoxyanthocyanidin Biosynthesis Pathway
Source: G3 (Bethesda). 2016 Mar 17;6(5):1439–47. doi: 10.1534/g3.115.026104 (PMC4856094; doi:10.1534/g3.115.026104)
Supplement: Supplemental Material [file supp_6_5_1439__index.html]

The Sorghum Gene for Leaf Color Changes upon Wounding (P) Encodes a Flavanone 4-Reductase in the 3-Deoxyanthocyanidin Biosynthesis Pathway — Supplemental Material 

# The Sorghum Gene for Leaf Color Changes upon Wounding (*P*) Encodes a Flavanone 4-Reductase in the 3-Deoxyanthocyanidin Biosynthesis Pathway

## Supplemental Material for Kawahigashi *et al.*, 2016

**Files in this Data Supplement:**

- Figure S1 - SDS-PAGE of time course of detection of Sb06g029550 protein in Figure 3C and detection of the Sb06g029550 protein in various sorghum in Figure 4A. (.pdf, 65 KB)
- Figure S2 - The repeat sequence and flanking sequences of a large insert in the Sb06g029550 allele in accessions JP501 and JP43800. (.pdf, 12 KB)
- Figure S3 - Expression of genes associated with secondary metabolism of 3-deoxyanthocyanidins, anthocyanidins or flavones in Nakei-MS3B. (.pdf, 19 KB)
- Figure S4 - Surface model of sorghum Sb06g029550 protein. (.pdf, 67 KB)
- Table S1 - Primers used in our study (except for the data shown in Figure 6). (.pdf, 34 KB)
- Table S2 - Primers used for gene expression analysis shown in Figure 3 and 6. (.pdf, 35 KB)
