# Supplementary material for: The Sorghum Gene for Leaf Color Changes upon Wounding (P) Encodes a Flavanone 4-Reductase in the 3-Deoxyanthocyanidin Biosynthesis Pathway
Source: G3 (Bethesda). 2016 Mar 17;6(5):1439–47. doi: 10.1534/g3.115.026104 (PMC4856094; doi:10.1534/g3.115.026104)
Supplement: Supplemental Material [file supp_g3.115.026104_FigureS1.pdf]

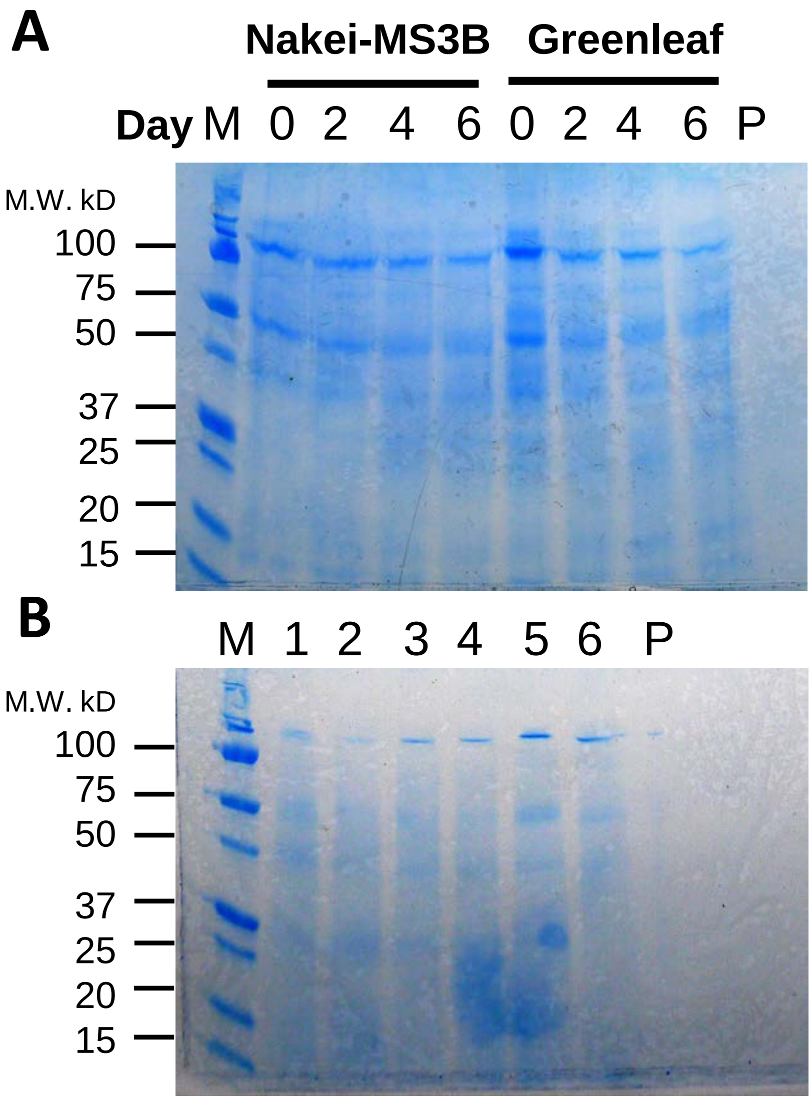

Figure S1

SDS-PAGE of time course of detection of Sb06g029550 protein in Figure 3C and detection of the Sb06g029550 protein in various sorghum in figure 4A.

Leaf strips from Nakei-MS3B and Greenleaf incubated on agar plate and the extracted protein were used for western blotting (figure 3C). M, protein standard marker; P, recombinant protein (positive control). (B) Soluble protein of leaf strips 6 days after cutting were used (figure 4A). Four accessions with the tan phenotype obtained from the NIAS sorghum core collection are marked as 1, JP43764; 2, JP43800 3, JP588; 4, JP501; in addition to 5, Greenleaf; 6, Nakei-MS3B; P, recombinant protein (positive control); M, protein standard marker.
