# Supplementary material for: The Sorghum Gene for Leaf Color Changes upon Wounding (P) Encodes a Flavanone 4-Reductase in the 3-Deoxyanthocyanidin Biosynthesis Pathway
Source: G3 (Bethesda). 2016 Mar 17;6(5):1439–47. doi: 10.1534/g3.115.026104 (PMC4856094; doi:10.1534/g3.115.026104)
Supplement: Supplemental Material [file supp_g3.115.026104_FigureS2.pdf]

Sb06g029550

452

↓

...CTGCTAGGCGACGGA**CATGGGCATG****GCGAAATGTTCAGATCTCCC......**

Repeat sequence

...... (unknown, no homology) ......

......CACCCTGAGGCACCATGTCCGCGTTGCATGGGCATGTGCTGGAC...

Repeat sequence

Figure S2

The repeat sequence and flanking sequences of a large insert in the Sb06g029550 allele in accessions JP501 and JP43800. The insert found at 452 bp (mRNA numbering) was not amplified by genomic PCR, which suggested that its size is >4 kbp.
