# Supplementary material for: The Sorghum Gene for Leaf Color Changes upon Wounding (P) Encodes a Flavanone 4-Reductase in the 3-Deoxyanthocyanidin Biosynthesis Pathway
Source: G3 (Bethesda). 2016 Mar 17;6(5):1439–47. doi: 10.1534/g3.115.026104 (PMC4856094; doi:10.1534/g3.115.026104)
Supplement: Supplemental Material [file supp_g3.115.026104_FigureS3.pdf]

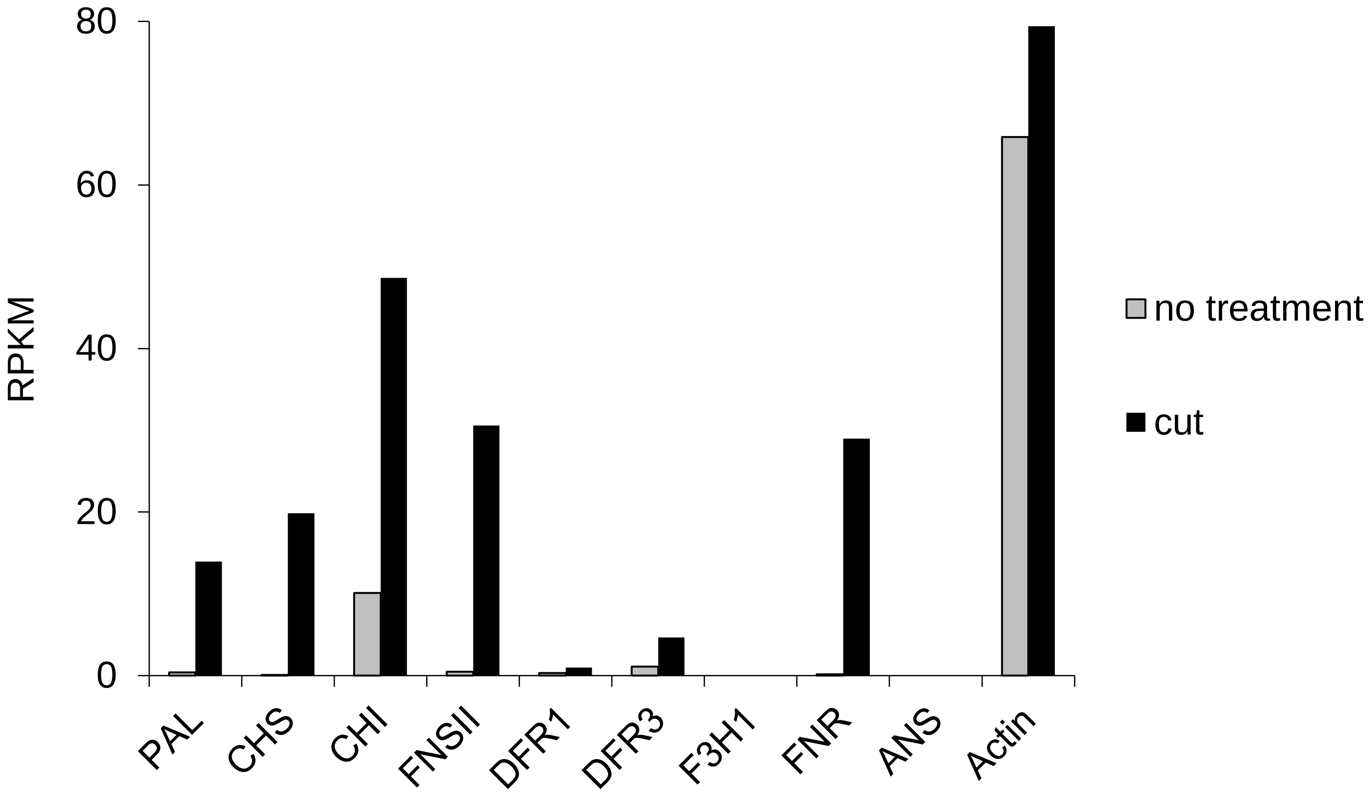

Figure S3

Expression of genes associated with secondary metabolism of 3-deoxyanthocyanidins, anthocyanindins or flavones in Nakei-MS3B. Expression levels were compared by mRNA-seq experiment in no treatment- and cut leaf strips on day 4 incubated on agar plate (Mizuno et al. 2014). RPKMs of genes encoding phenylalanine ammonia lyase (PAL), chalcone synthase (CHS), and chalcone isomerase (CHI), flavone synthase II (FNSII), dihydroflavonol 4-reductases (DFRs), flavanone 3-hydroxylases (F3H), flavanone 4-reductase (FNR), or anthocyanindin synthase (ANS) are shown. RPKMs of actin genes are also shown as control.
