# Supplementary material for: The Sorghum Gene for Leaf Color Changes upon Wounding (P) Encodes a Flavanone 4-Reductase in the 3-Deoxyanthocyanidin Biosynthesis Pathway
Source: G3 (Bethesda). 2016 Mar 17;6(5):1439–47. doi: 10.1534/g3.115.026104 (PMC4856094; doi:10.1534/g3.115.026104)
Supplement: Supplemental Material [file supp_g3.115.026104_FigureS4.pdf]

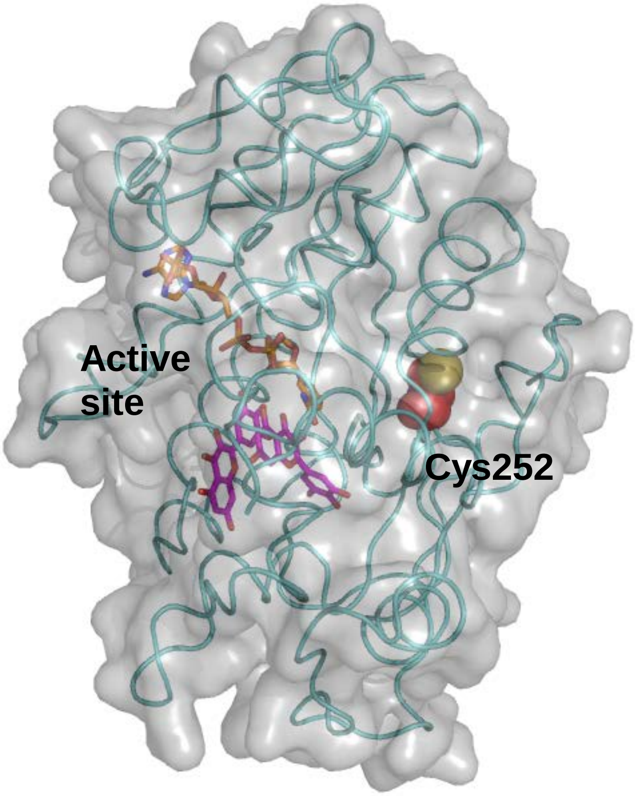

Figure S4

Surface model of sorghum Sb06g029550 protein. The model was created with the SWISS-MODEL web server (Arnold *et al.* 2006) by homology modeling on the basis of the crystal structure of grape dihydroflavonol 4-reductase (Protein Data Bank code 3C1T; Trabelsi *et al.* 2008). The C_α trace is shown in light blue. Cys252 is shown as a spherical model. Quercetin (magenta) and NADP (orange) molecules, found in the reference model, are superimposed and shown as stick models.
