# Supplementary material for: The Sorghum Gene for Leaf Color Changes upon Wounding (P) Encodes a Flavanone 4-Reductase in the 3-Deoxyanthocyanidin Biosynthesis Pathway
Source: G3 (Bethesda). 2016 Mar 17;6(5):1439–47. doi: 10.1534/g3.115.026104 (PMC4856094; doi:10.1534/g3.115.026104)
Supplement: Supplemental Material [file supp_g3.115.026104_TableS1.pdf]

Table S1. Primers used in our study (except for the data shown in Figure 6).

**Genetic mapping**			
SB25789	forward	5'-TTTTCGCGTGGTAAGATCTAGAGTA-3'	SSR marker
	reverse	5'-TCCCCCATGACATAAAATCCACTC-3'	
CA24920	forward	5'-TACAAGTTTGAAGAGCTTGCTCAG-3'	CAPS *Eco* RI
	reverse	5'-ACAATCTGAGTTTGGAAGGGTTC-3'	
SB25792	forward	5'-TGGGCAACCAAATTACATCCTTCT-3'	SSR marker
	reverse	5'-GGTTGGGCCCTTGAAAATAAGAGA-3'	
CA29510	forward	5'-TAAATCAGTTTGTGCATGACCG-3'	In-del marker, CAPS *Dra* I
	reverse	5'-GAACAATGTGACCAGCGAACG-3'	
CA29530	forward	5'-GTACCAACGCAGTCTGCTCAC-3'	SNP marker
	reverse	5'-CAGAAGTGCAAATCTTTTCTTCAAC-3'	
Other primers			Yonemaru et al. 2009
**RT-PCR and qRT-PCR for candidate gene analysis**			
Sb06g029540	forward	5'-caccTGCGTTGGTGGGCGCCGGCGATG-3'	
(ORF1)	reverse	5'-CATGGCTTGTACTAGTTTAAATCA-3'	
Sb06g029550	forward	5'-caccTGCGTTGGTGTGGCTGGCAATATG-3'	
(ORF2)	reverse	5'-GCTAAATCGGGCATCTATATATCA-3'	
Sb06g029560	forward	5'-caccATGTCGTCGTCCGTGGTCGAAAAG-3'	
(ORF3)	reverse	5'-GGGCTACACATCAGACTATCCACG-3'	
Sb06g029570	forward	5'-caccATGTCAGAGGGCGGCAGGAAGCAG-3'	
(ORF4)	reverse	5'-TCGCTTTGCAATGCAAAGAAAGTA-3'	
SbActin	forward	5'-TTCCAGCAGATGTGGATCTCCAAG-3'	
(Sb03g040880)	reverse	5'-ATGTTTCTTCATGTAGAACATCGAT-3'	
**Genomic PCR of Sb06g029550**			
1F	forward	5'-AATCATCAGGCCTGCTGCTAGACT-3'	
1R	reverse	5'-AAGACGTAATCGCAGCCGGCGACA-3'	
2F	forward	5'-TTGCTTATAGAAAAAGAATTGAC-3'	
2R	reverse	5'-AGAATGGGTGCAGAATCATCGGA-3'	
3F	forward	5'-AACACAATGAGGTCGTGCGTGAA-3'	
3R	reverse	5'-TGGTATCTGCTGCAAATGTACCT-3'	
4F	forward	5'-ATTGCTGATTATCTTCAGGCGA-3'	
4R	reverse	5'-AAAATGAATAGCGAGAACGCAAA-3'	
**RT-PCR of a fragment encoding full-length Sb06g029550**			
Sb06g029550	forward	5'-ATGAAGACGGCGTGCGTTACTG-3'	
	reverse	5'-AGCTCTAGATCAGTAGGGCAGAATTCCTAA -3'	
