# Supplementary material for: The Sorghum Gene for Leaf Color Changes upon Wounding (P) Encodes a Flavanone 4-Reductase in the 3-Deoxyanthocyanidin Biosynthesis Pathway
Source: G3 (Bethesda). 2016 Mar 17;6(5):1439–47. doi: 10.1534/g3.115.026104 (PMC4856094; doi:10.1534/g3.115.026104)
Supplement: Supplemental Material [file supp_g3.115.026104_TableS2.pdf]

Table S2. Primers used for gene expression analysis shown in Figure 3 and 6.

Gene number	Protein		Sequence	Reference
Sb06g029550	FNR	forward	5'-GGGTAACAAGAAGACGATGAAGA-3'	This study
		reverse	5'-CTGGATCCTGTGCCTCGAAGT-3'	
Sb05g020220	CHS1	forward	5'-CTGGACCAGGTGGAGGCCAAGG-3'	Liu et al. 2010
		reverse	5'-TAGACAGGACCTGTCTACCA-3'	
Sb01g003330	CHI	forward	5'-AAGTTCAAGGAGGCGTTCAA-3'	Liu et al. 2010
		reverse	5'-CGACTGGCTGGTTCTCTTTC-3'	
Sb06g031790	F3H1	forward	5'-CCGACTGCCAAGGAATTCGC-3'	Liu et al. 2010
		reverse	5'-CACACGCGACGATATAAACC-3'	
not assigned	F3H2	forward	5'-AGCAGAGTGGCAAGGAGTTC-3'	Liu et al. 2010
		reverse	5'-CACACGCGACGATATAAACC-3'	
Sb03g028880	DFR1	forward	5'-CTCGATCCTGAAGCAGGTGC-3'	Liu et al. 2010
		reverse	5'-CACGAGAACACTATGAGCTT-3'	
Sb04g004290	DFR3	forward	5'-CTCGATCCTGAAGCAGGTGC-3'	Liu et al. 2010
		reverse	5'-ACTAATTGCCGCATCTGTCC-3'	
Sb04g000260	ANS	forward	5'-CTCCATCCCGCTCGAGTA-3'	Liu et al. 2010
		reverse	5'-CTCCTTTTCCTGGATGGGGA-3'	
Sb02g000220	FNSII	forward	5'-CGCAAGACCACCGTCTTCTT-3'	Du et al. 2010
		reverse	5'-GGTAGCTTTTCCTGTTGCCG-3'	
Sb06g022740	PAL	forward	5'-ACGACGACCCCTGCAGCGCCAACT-3'	Kawahigashi et al. 2011
		reverse	5'-AGCAGATGGGCAGGGGCTTGCCGT-3'	
Sb03g040880	Actin	forward	5'-TTCCAGCAGATGTGGATCTCCAAG-3'	Kawahigashi et al. 2011
		reverse	5'-ATGTTTCTTCATGTAGAACATCGAT-3'	
